# Chlamydia trachomatis homotypic inclusion fusion is promoted by host microtubule trafficking

**DOI:** 10.1186/1471-2180-13-185

**Published:** 2013-08-07

**Authors:** Theresa S Richards, Andrea E Knowlton, Scott S Grieshaber

**Affiliations:** 1 Department of Oral Biology, College of Dentistry, University of Florida, Box 100424, Gainesville, FL 32610, USA

**Keywords:** Chlamydia, Dynein, Microtubules, Vesicle fusion, Centrosomes

## Abstract

**Background:**

The developmental cycle of the obligate intracellular pathogen *Chlamydia* is dependant on the formation of a unique intracellular niche termed the chlamydial inclusion. The inclusion is a membrane bound vacuole derived from host cytoplasmic membrane and is modified significantly by the insertion of chlamydial proteins. A unique property of the inclusion is its propensity for homotypic fusion. The vast majority of cells infected with multiple chlamydial elementary bodies (EBs) contain only a single mature inclusion. The chlamydial protein IncA is required for fusion, however the host process involved are uncharacterized.

**Results:**

Here, through live imaging studies, we determined that the nascent inclusions clustered tightly at the cell microtubule organizing center (MTOC) where they eventually fused to form a single inclusion. We established that factors involved in trafficking were required for efficient fusion as both disruption of the microtubule network and inhibition of microtubule trafficking reduced the efficiency of fusion. Additionally, fusion occurred at multiple sites in the cell and was delayed when the microtubule minus ends were either no longer anchored at a single MTOC or when a cell possessed multiple MTOCs.

**Conclusions:**

The data presented demonstrates that efficient homotypic fusion requires the inclusions to be in close proximity and that this proximity is dependent on chlamydial microtubule trafficking to the minus ends of microtubules.

## Background

*Chlamydia trachomatis* causes sexually transmitted infections and is the leading cause of preventable blindness worldwide [1]. *Chlamydia* are Gram-negative, obligate intracellular bacteria with a unique, biphasic developmental cycle that takes place in a membrane-bound vacuole termed the inclusion. The infectious but metabolically inactive elementary body (EB) attaches to epithelial cells and initiates its uptake through parasite mediated endocytosis [2]. Once internalized, EBs differentiate into metabolically active but non-infectious reticulate bodies (RBs) which replicate by binary fission. As the infection progresses, RBs differentiate into EBs in an asynchronous manner and these infectious EBs are eventually released into the host to initiate a additional rounds of infection.

Following infection, the inclusion membrane is modified through the insertion of multiple bacterial type three secreted effector proteins [3]. These inclusions are non-fusogenic with the endosomal and lysosomal pathways [4]. Inclusions are trafficked along microtubules in a dynein-dependent manner to the microtubule organizing center (MTOC) where they intercept host-derived lipids to maintain the integrity of the expanding inclusion [5]. Thus, despite being sequestered within a membrane-bound vacuole, chlamydiae manipulate the host and subvert host pathways to establish an environment that is not only conducive to replication and differentiation but also simultaneously protected from host immune responses.

At high multiplicities of infection, multiple inclusions fuse into a single inclusion. This fusion event is critical for pathogenicity; rare isolates with non-fusogenic inclusions are clinically associated with less severe signs of infection and lower numbers of recoverable bacteria than wild-type isolates [6]. Inclusion fusion occurs even between different *C. trachomatis* serovars potentially facilitating genetic exchange between serovars [7]. Previous studies have demonstrated that the fusion of chlamydial inclusions requires bacterial protein synthesis and is inhibited during growth at 32°C [8]. Specifically, the inclusion membrane protein IncA is required for the homotypic fusion of chlamydial inclusions [9].

The importance of both inclusion trafficking and inclusion fusion have been established but the role that inclusion trafficking plays in promoting fusion has not been investigated. In this study we demonstrate that inclusion migration along microtubules promotes inclusion fusion. Interestingly, although this dynein dependent migration was required for the normal timing of inclusion fusion, inhibition of this trafficking was eventually overcome later during infection.

## Methods

### Organisms and cell culture

All cells were obtained from the American Type Culture Collection. Cell lines are: McCoy (McCoy B, CRL-1696), HeLa (HeLa 229, CCL-2.1), Cos7 (COS-7, CRL-1651) and neuroblastoma (N1E-115, CRL-2263). *Chlamydia trachomatis* serovars are: L2 (LGV 434), G (UW-524/CX) and J (UW-36/CX). *C. trachomatis* were propagated in McCoy or HeLa cells. EBs were purified by Renografin (Bristol-Myers Squibb, New York, NY, USA) density gradient centrifugation as previously described [10,11]. HeLa and Cos7 cells were grown in RPMI-1640 (Lonza, Basel, Switzerland) supplemented with 10% FBS (Gibco/Life Technologies, Grand Island, NY, USA) and 10 μg/mL gentamicin (Gibco). McCoy and neuroblastoma cells were grown in DMEM (Lonza) supplemented with 10% FBS (Gibco) and 10 μg/mL gentamicin (Gibco). All cells were grown in 5% CO_2_ at 37°C.

### Infections

All infections were carried out as follows unless otherwise noted. Cells were incubated with *C. trachomatis* EBs in Hank’s balanced salt solution (HBSS) (Invitrogen/Life Technologies, Grand Island, NY, USA) for 30 min at 22°C. The inoculum was replaced with prewarmed, 37°C, complete media. For nocodazole treated cells, the inoculum was replaced with prewarmed, 37°C, complete media containing 5 μg/mL nocodazole. Infected cells were incubated in 5% CO_2_ at 37°C.

### Synchronized infections

Cells were incubated with *C. trachomatis* EBs in HBSS (Invitrogen) at MOI = 1000 for 5 min at 22°C. The cells were washed three times with HBSS plus 100 μg/mL heparin (Pharmacia, Peapack, NJ, USA) and twice with HBSS without heparin. Prewarmed, 37°C, complete media was added and infected cells were incubated in 5% CO_2_ at 37°C.

### Transfections and plasmids

HeLa cells were grown on 12 mm number 1.5 borosilicate glass coverslips coated with Poly-L-lysine (Sigma-Aldrich, St. Louis, MO, USA) to obtain a monolayer of approximately 65% confluency. Transfections were carried out using Lipofectamine 2000 (Invitrogen) according to the manufacturer’s instructions. Expression from the transfected vectors was allowed to proceed for at least 24 h prior to experimentation. Expression vectors used were pEGFP-C3 (Clontech, Mountain View, CA, USA), EB1-GFP and EB1.84-GFP. The EB1-GFP plasmid was a kind gift from Dr Jennifer S. Tirnauer, University of Connecticut Health Center. The EB1.84-GFP plasmid was generated by PCR cloning of the N terminal end of EB1 and cloning into pDest-NGFP as described by Askham et al. [12].

### Micro-injections

Cos7 cells were grown on 25 mm number 1.5 borosilicate glass coverslips coated with Poly-L-lysine (Sigma-Aldrich) to obtain a monolayer of approximately 50% confluency. Micro-injection was performed using an automated system described previously [5]. Cells were injected with either mouse monoclonal antibody to dic74.1 (Covance, Princeton, NJ, USA) or antiCD80 (Invitrogen). Following injection, cells were washed once with prewarmed, 37°C, complete media, and fresh prewarmed media was added. Approximately 10–15 min after injection, the cells were infected with *C. trachomatis* and incubated in 5% CO_2_ at 37°C. The cells were fixed with 4% paraformaldehyde and permeabilized with 0.5% TritonX 100. The injected antibodies were detected using AlexaFluor 488-conjugated goat anti-mouse IgG (Molecular Probes/Life Technologies, Grand Island, NY, USA).

### Antibodies and microscopy

For fluorescent antibody staining, infected cells were fixed with cold methanol for 10 min. Antibodies used in these experiments were mouse monoclonal anti-γ-tubulin (Sigma-Aldrich), anti-chlamydial inclusion membrane protein IncA a gift from Dr. Dan Rockey, at the Oregon State University, and anti-chlamydial MOMP a gift from Dr. Harlan Caldwell, Rocky Mountain Labs NIAID. *C. trachomatis* was stained with human serum (Sigma-Aldrich) unless otherwise noted. To visualize the primary antibodies, cells were incubated with the appropriate AlexaFluor conjugated secondary antibody: 488, 567 or 647 against mouse, rabbit or human IgG (Molecular Probes). To visualize DNA, cells were stained with the far-red fluorescent dye DRAQ5 (Biostatus Limited, Leicestershire, UK). Images were acquired using a spinning disk confocal system connected to a Leica DMIRB microscope with a 63× oil-immersion objective, equipped with a Photometrics cascade-cooled EMCCD camera, under the control of the Open Source software package μManager (http://www.micro-manager.org/). Images were processed using the image analysis software ImageJ (http://rsb.info.nih.gov/ij/). Projections were constructed using the ImageJ image software (Wayne Rasband, U.S. National Institutes of Health, http://rsb.info.nih.gov/ij).

## Results

### Inclusion fusion occurs at the MTOC

**Figure 1:**
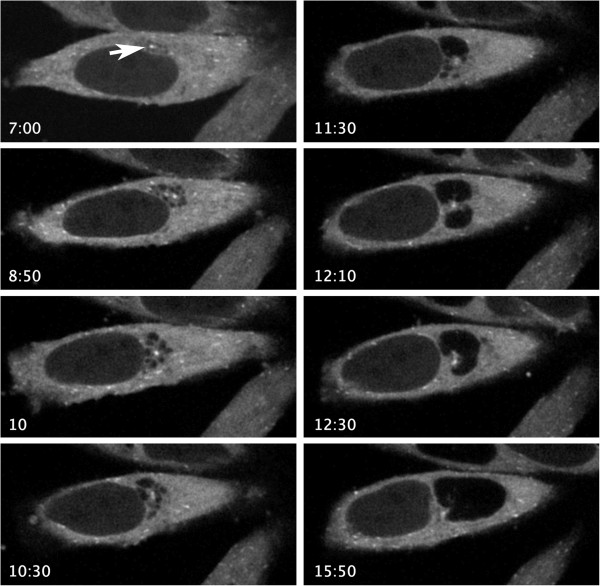
**Inclusion fusion occurs at the centrosomes.** HeLa cells were transfected with EB1-GFP to visualize centrosomes (arrow in A). Eighteen hours post-transfection, cells were infected with C. trachomatis at MOI = 20. During infection, cells were photographed every 10 minutes until 24 hpi. Times post infection are indicated in each corresponding image.

The location and dynamics of inclusion fusion are currently poorly understood. To determine the subcellular location of fusion in multiply infected cells, HeLa cells were transfected with EB1-GFP. EB1 is a microtubule end plus end tracking protein and serves to identify the site of the microtubule organizing center (MTOC). Eighteen hours post-transfection, cells were infected with *C. trachomatis* at MOI ~20. Infected cells were imaged every 10 minutes for a total of 24 hours. Representative time points (Figure 1) revealed that early during infection, multiple inclusions were present adjacent to cell centrosomes (Figure 1, 8:50–11:30 hpi). As the infection proceeded, fusion occurred between closely grouped inclusions (Figure 1, 11:30–12:30 hpi). Fusion continued until there was a single inclusion (Figure 1, 12:30 hpi) which continued to expand as the developmental cycle progressed (Figure 1, compare 12:30 and 15:50 hpi). In these experiments, fusion was only observed between inclusions tightly clustered around the MTOC/centrosome of the host cell. (Also see Additional file 1: Movie 1).

### Intact microtubules are required for efficient inclusion fusion

**Figure 2:**
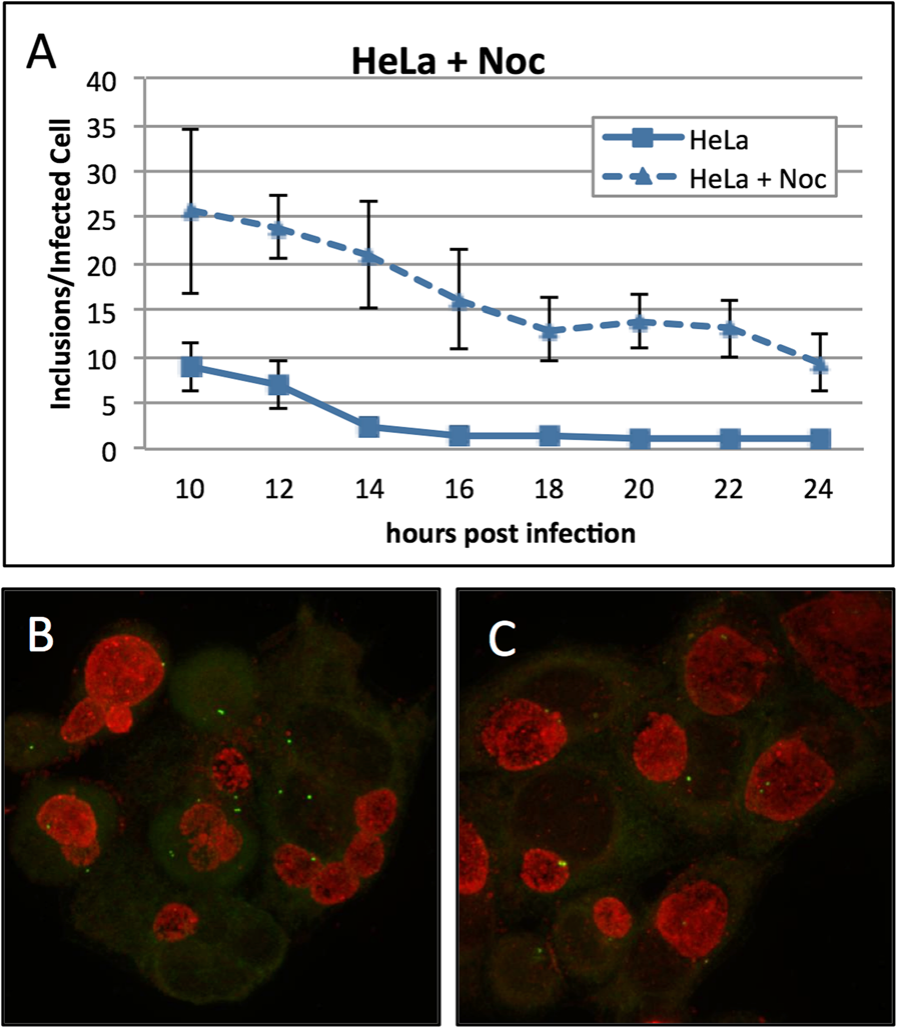
**Inclusion fusion is delayed in HeLa cells treated with nocodazole.** HeLa cells were infected with *C. trachomatis* at MOI ~ 9 in the presence and absence of nocodazole (Noc) and fixed at 10, 12, 14, 16, 20, 22 and 24 hpi. Cells were stained with human sera and anti-g-tubulin antibodies and inclusions were enumerated **(A)**. Representative treated and untreated HeLa cells **(B** and **C,** respectively**)**.

We demonstrated that fusion occurs at the centrosomes and we have previously reported that trafficking on microtubules is required for the localization of chlamydial inclusions at the centrosomes. We asked whether the microtubule network influenced inclusion fusion. HeLa cells were infected with *C. trachomatis*. Following infection, cells were incubated in the presence or absence of nocodazole and then fixed every two hours between 10 and 24 hpi. Inclusion fusion occurred at approximately 14 hpi for untreated cells (Figure 2A). In cells that had been treated with nocodazole, fusion was significantly delayed. Nocodazole-treated cells had an average of eight inclusions per cell at 24 hpi (Figure 2A). Fusion was not completely abolished by nocodazole treatment suggesting that the fusion machinery does not require microtubules but instead that the microtubules accelerate fusion. Representative pictures of nocodazole treated and untreated cells are shown in Figure 2B and C, respectively.

### Inhibiting dynein function in HeLa cells inhibits inclusion fusion

**Figure 3:**
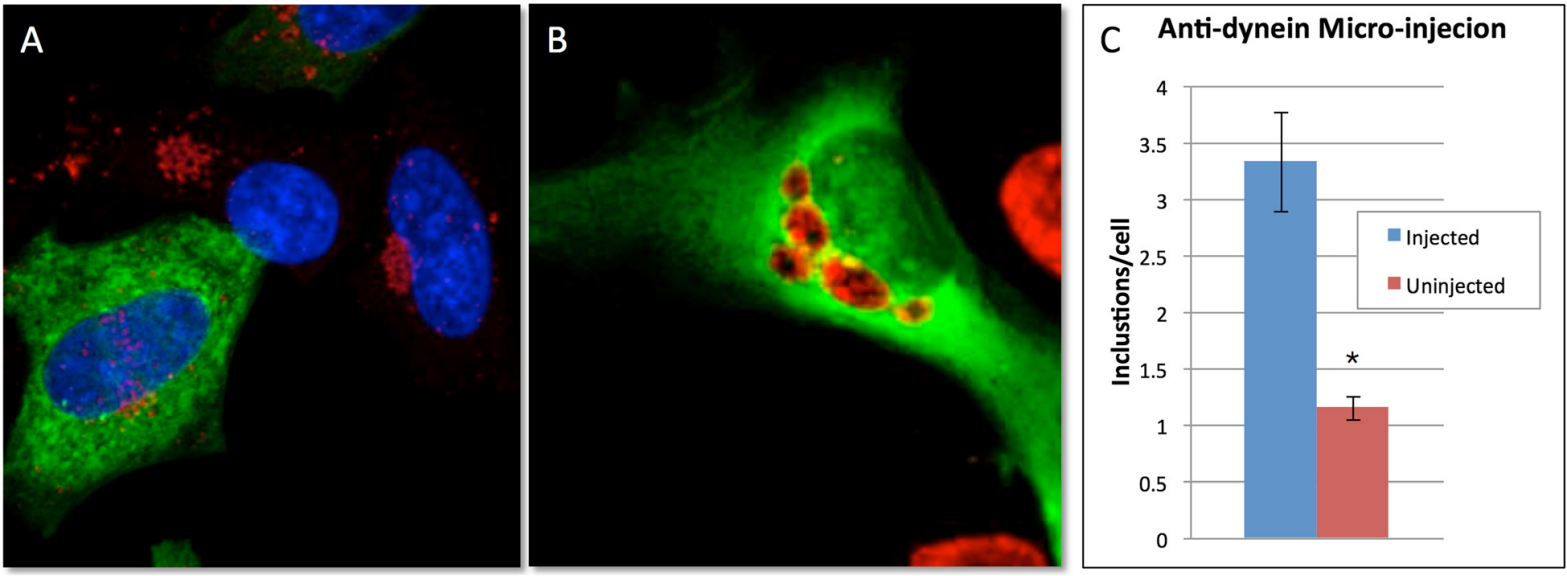
**Chlamydial inclusion trafficking and fusion is dynein dependent.** Cos7 cells were infected with *C. trachomatis* serovar L2 following micro-injection with anti-dynein antibodies. Uninjected cells were infected in parallel. Twenty-four hours postinfection, cells were fixed and stained with human sera (red) and the appropriate secondary for the anti-dynein antibody (green). Representative picture of anti-dynein injected cells at 6 and 24 hpi **(A** and **B,** respectively**)**. Inclusions per infected cell were enumerated for injected and uninjected cells at 24 hpi, P < 0.0001 **(C)**.

Chlamydial microtubule trafficking is dependent on the host microtubule motor protein dynein. To investigate the role of dynein in inclusion fusion, we injected Cos7 cells with anti-dynein intermediate chain antibodies (DIC74.1). Following injection, cells were infected with *C. trachomatis*. Uninjected cells were infected in parallel. Cells were fixed at 6 and 24 hpi. In cells that had been injected with anti-dynein antibodies, inclusion clustering was decreased early in infection and inclusion fusion decreased (Figure 3A and B, respectively). At 24 hpi, there was a significant difference between injected and uninjected cells (P < 0.001); injected cells averaged three inclusions per infected cell while uninjected cells averaged one inclusion per infected cell (Figure 3C).

### Fusion is delayed in neuroblastoma cells

**Figure 4:**
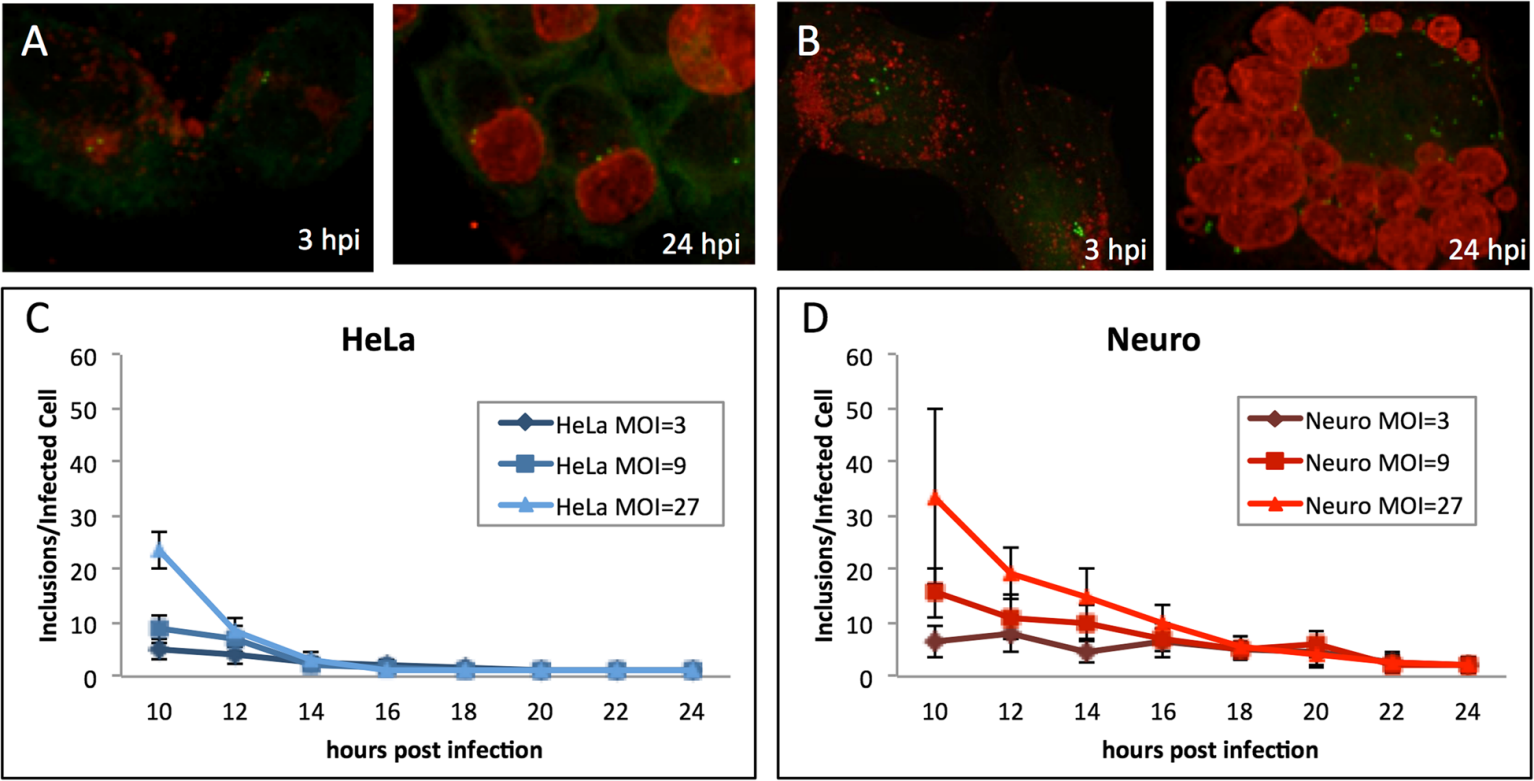
**Inclusion fusion is delayed in cells with multiple unclustered centrosomes.** HeLa cells **(A)** and neuroblastomas **(B)** were infected with *C. trachomatis* at MOI ~ 27 and fixed at 3 and 24 hpi. Cells were stained with anti-g-tubulin antibodies (green) and human sera (red). HeLa cells **(C)** and neuroblastomas **(D)** were infected with *C. trachomatis* at MOI ~ 3, 9 and 27 and fixed at 10, 12, 14, 16, 20, 22 and 24 hpi. Cells were stained with human sera and inclusions were enumerated.

We established that inclusion fusion occurs at cell centrosomes and both dynein and microtubules promote fusion. We next asked whether infection of cells with multiple centrosomes would lead to multiple sites of fusion. The mouse neuroblastoma cell line N115 has significant centrosome number defects containing an average of eight centrosomes per cell [13,14]. This allowed us to ask whether defects in centrosome numbers would affect inclusion fusion. HeLa and neuroblastoma cells were infected with *C. trachomatis* at three different multiplicities of infection. Infections were fixed at 3 hpi and every two hours between 10 and 24 hpi. Early inclusions were present near the tightly clustered centrosomes in HeLa cells but in neuroblastoma cells, which have multiple centrosomes distributed throughout the cell, early inclusions were present throughout the host cytosol clustered at the scattered centrosomes (Figure 4A 3 hpi and 4B 3 hpi, respectively). At 24 hpi, infected HeLa cells had a single inclusion adjacent to the centrosomes (Figure 4 24 hpi). While some infected neuroblastoma cells had single inclusions at 24 hpi, infected neuroblastoma cells could still be found with multiple unfused inclusions (Figure 4B 24 hpi). In infected HeLa cells, fusion of chlamydial inclusions occurred at approximately 12-14 hpi (Figure 4C). Fusion was delayed in neuroblastoma cells, occurring at approximately 16-18 hpi (Figure 4D).

### Neuroblastoma cells are fusion competent and inclusion membrane protein IncA is present on their inclusion membranes

**Figure 5:**
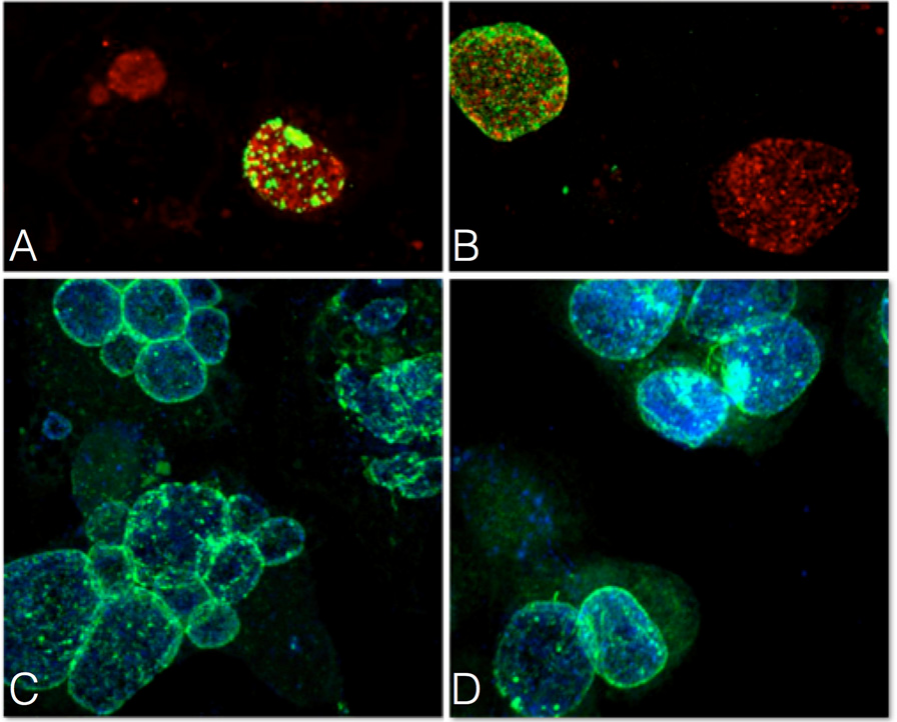
**Neuroblastomas are fusion competent and IncA localizes to the inclusion membrane during infection.** HeLa cells **(A)** and neuroblastomas **(B)** were infected with *C. trachomatis* serovar G. At 40 hpi, cells were superinfected with *C. trachomatis* serovar L2 and fixed four hours after superinfection. Cells were stained with human sera (red) and anti-L2 MOMP antibodies (green). HeLa cells **(C)** and neuroblastomas **(D)** were infected with *C. trachomatis* serovar L2 at MOI ~ 9 and fixed 24 hpi. Cells were stained with human sera (blue) and anti-IncA antibodies (green).

In order to determine whether neuroblastomas were fusion competent, HeLa and neuroblastoma cells were serially infected with different *C. trachomatis* serovars. Cells were infected with *C. trachomatis* serovar G for 40 hours and then superinfected with *C. trachomatis* serovar L2 for four hours. In both HeLa cells and neuroblastomas, fusion occurred between inclusions containing G and L2 indicating that the inclusions in neuroblastoma cells are fusion competent (Figure 5A and 5B). The inclusion membrane protein IncA is required for inclusion fusion and delays in IncA membrane localization lead to delayed homotypic fusion [8,9,15]. Therefore, we assessed the location of IncA in the infected neuroblastoma cells. HeLa and neuroblastoma cells were infected with *C. trachomatis* serovar L2, fixed at 24 hpi and stained with antibodies to IncA. IncA was present on inclusion membranes in both HeLa and neuroblastoma cells (Figure 5C and 5D, respectively). Taken together, these data demonstrate that the delay in inclusion fusion observed in neuroblastoma cells is not due to differences in fusion competency or to differences in the presence of IncA. Additionally, when infected neuroblastomas were grown on fibronectin micropatterns to force centrosome clustering, inclusion fusion was restored (Additional file 2: Figure S1).

### Fusion is delayed in cells with unanchored microtubule minus ends

**Figure 6:**
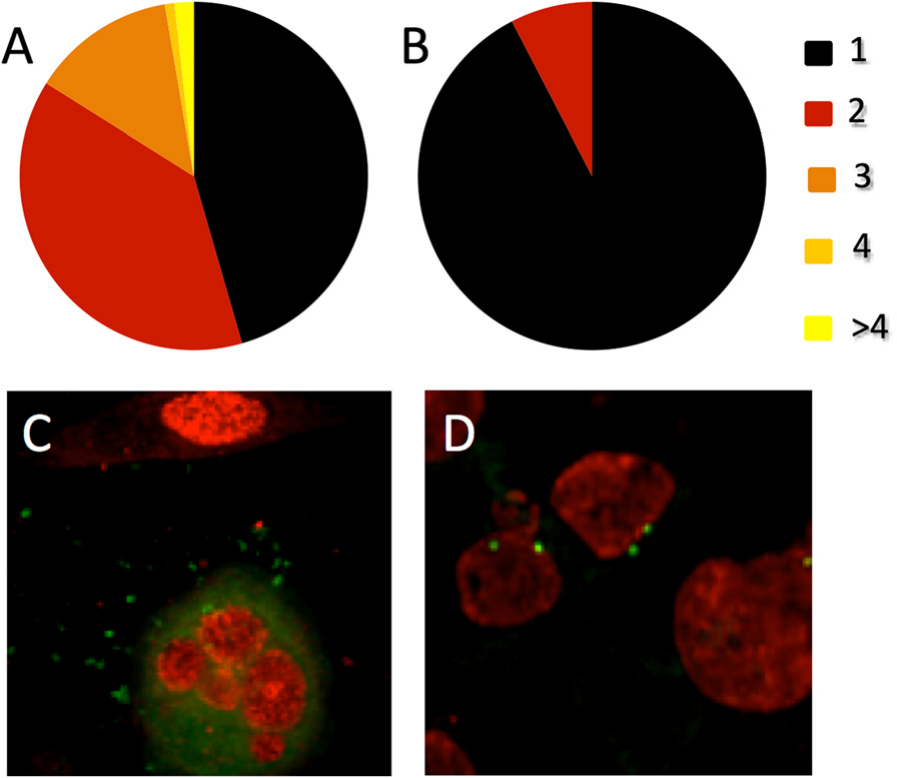
**Transfection with EB1.84-GFP disrupts inclusion fusion.** HeLa cells were transfected with EB1.84-GFP or mock transfected. They were then infected with *C. trachomatis*. Twenty-four hours postinfection, cells were fixed and stained with human sera and inclusions per infected cell were enumerated. The distribution in the number of inclusions per infected cell is shown for the EB1.84-GFP transfected and mock transfected cells in **A** and **B**, respectively. Mock transfected cells were also stained with anti-g-tubulin antibodies (green). Representative transfected and mock transfected cells shown in **C** and **D**, respectively.

Chlamydial inclusion fusion occurs at host centrosomes and is delayed when extra centrosomes are present. Inclusion migration is unidirectional resulting in the chlamydial inclusion residing at the cell centrosome for its entire intracellular growth phase. In the cell, the centrosome acts as the organizing center that anchors the majority of microtubule minus ends. We hypothesize that inclusion fusion is promoted by inclusion crowding at the anchored minus ends of microtubules. To determine if fusion is dependent on microtubule minus end anchoring, we transfected HeLa cells with the GFP tagged EB1 mutant, EB1.84-GFP. Cells expressing EB1.84-GFP have defects in microtubule organization and centrosomal anchoring resulting in unanchored free microtubule minus ends [12]. When we compared inclusion fusion in the cells that had been mock transfected to cells transfected with EB1.84-GFP, the EB1.84 producing cells were markedly delayed in inclusion fusion. At 24 hpi, transfected cells averaged 1.7 inclusions per infected cell while mock transfected cells averaged one inclusion per infected cell (P < 0.001). We also quantitated the distribution of inclusion numbers in these cells, slightly under half of the cells transfected with EB1.84-GFP contained one inclusion (46%) while the majority of mock transfected cells (92%) had a single inclusion (Figure 6A and B, respectively). Additionally, many of the EB1.85 transfected cells had four or more inclusions per cell, while mock transfected cells never had more than two inclusion per cell (Figure 6A and B, respectively). Representative images of inclusions in transfected and mock transfected cells are shown in Figure 6C and D, respectively.

## Discussion and conclusion

The ability of *C. trachomatis* inclusions to fuse is critical to pathogenicity. Compared to wild type strains, rare isolates with non-fusogenic inclusions are clinically associated with less severe signs of infection and lower numbers of recoverable bacteria [6]. In cell culture however, a role for inclusion fusion has yet to be determined. Matched pairs of non-fusing and fusing strains as well as nocodazole treated and untreated matched sets grow at similar rates and produce comparable numbers of progeny [16,17].

Chlamydial inclusion fusion is however critical to pathogenicity though the exact reason for this remains elusive. Homotypic inclusion fusion in *C. trachomatis* is a phenotype shared by all serovars. Considering that the metabolically active form of this obligate intracellular organism is spatially sequestered, it is plausible that sharing a single inclusoplasm facilitates genetic and/or nutrient exchange between between co-infecting trachomatis serovars thus promoting their fitness within a population. It is well established that *C. trachomatis* stores sugars in the form of glycogen in the inclusion [18,19] and this glycogen storage is linked to virulence as loss of the chlamydial cryptic plasmid results in both loss of glycogen storage as well as reduced virulence [20]. Homotypic inclusion fusion would allow this resource to be shared by bacteria and may lead to a competitive growth advantage in a hostile environment such as the reproductive track during in vivo infection.

A complete understanding of mechanisms and factors required for homotypic fusion is currently unknown. The chlamydial inclusion membrane protein IncA is the only chlamydial factor known to be required for homotypic inclusion fusion [9,21]. Additionally, no host factors have been identified to be required for homotypic fusion.

Here, we describe a novel role for proper inclusion trafficking in inclusion fusion. Through live cell imaging studies, we showed that inclusion fusion occurs predominantly at a single site within host cells. This site was invariably the MTOC of the cell (Figure 1 and Additional file 1). Early in infection, multiple inclusions cluster tightly at the MTOC and remain associated as these inclusions begin to fuse. After fusion is complete, the single inclusion retains its close association with the MTOC as it continues to expand.

The MTOC contains the cells centrosomes and acts as an organizing foci for the cell. Additionally, the MTOC acts as the nucleation point for cellular microtubules. Host microtubules are polymerized in a polar fashion; the plus ends undergo rapid polymerization while the minus ends are anchored at the MTOC which allows for directional transport along the microtubules. We previously demonstrated that the the nascent chlamydial inclusion trafficks along microtubules using the microtubule motor protein dynein [5]. This study demonstrates that inclusion migration is a critical component for efficient fusion as both the dynein motor protein and intact microtubules are important for inclusion fusion. The requirement for both an intact microtubule network and the dynein motor protein along with the observation that fusion takes place between closely adjacent inclusions suggests that migration to a central location in the cell is a mechanism to physically drive the inclusions together. This increases the likelihood that the fusogenic protein IncA on neighboring inclusions will interact, thereby enhancing a timely fusion. This hypothesis is further supported by the observation that when the minus ends of the microtubules are not anchored (EB1.84 expressing cells) or not anchored at a single site in the cell (neuroblastomas), fusion was severely delayed. Interestingly, in neuroblastoma cells, the non fused inclusions appear to be in close proximity to each other however the resolution of fluorescence microscopy cannot resolve molecular level interactions. This suggests that for the chlamydial fusion protein IncA to interact with an IncA protein on a second inclusion, the distance between them would likely need to be very small. Interestingly, fusion is only delayed under these circumstances suggesting that eventually multiple inclusions in the cell come in close enough contact for the IncA driven fusion system to mediate fusion.

Overall our data support a model where nascent chlamydia-containing inclusions traffic along microtubules using the dynein motor protein to directionally traffic to the minus ends of microtubules. If the minus ends of the microtubules are anchored at the MTOC, then the multiple inclusions make close contact and are spatially arranged to encourage fusion. Interestingly, this trafficking takes place prior to IncA expression. Inclusion migration is rapid and occurs within the first few hours of infection however IncA is only expressed during the mid cycle of chlamydial infection, about 8 hours after infection [22]. The inclusions are maintained in a fusion-supporting organization until fusion is initiated through IncA protein expression and insertion into the inclusion membrane. This suggests a stepwise pathway of establishing the mature, fusion-competent chlamydial inclusion.

We have shown that inclusion fusion occurs at host cell centrosomes and that in order for fusion to result in a single inclusion, nascent inclusions must be transported by dynein along intact, anchored microtubules to a single site. Comprehending the role of microtubule trafficking in inclusion fusion dynamics is crucial to a complete understanding of the mechanisms by which this obligate intracellular pathogen promotes its intracellular survival and pathogenicity.

## Authors’ contributions

TR carried out the infections and immunofluorescence experiments and drafted the manuscript. AK acquired confocal images and contributed to data analysis. SG contributed to data analysis and finalized the manuscript. All authors read and approved the final manuscript.

## Supplementary Material

Additional file 1 **Inclusion fusion occurs at minus ends of microtubules.** Movie of Figure 1. Click here for file

Additional file 2: Figure 2 Centrosome positioning affects chlamydial inclusion localization. Uninfected and infected neuroblastomas were plated on CYTOOchips (glass coverslips imprinted with fibronectin micropatterns). Each micropattern is indicated in the lower left of the top panel. Infected cells were fixed at 12 and 24 hpi (top and bottom panel for each shape, respectively). Cells were stained with antibodies to g-tubulin (green) and Chlamydia (red). Nucleic acid is visualized by staining with DRAQ5 (blue). Click here for file

## References

[B1] Weinstock H Berman S Cates W Sexually transmitted diseases among American youth: incidence and prevalence estimates, 2000 Perspect Sex Reprod Health 2004 36 6 10 10.1363/3600604 14982671

[B2] Clifton DR Fields KA Grieshaber SS Dooley CA Fischer ER Mead DJ Carabeo RA Hackstadt T A chlamydial type III translocated protein is tyrosine-phosphorylated at the site of entry and associated with recruitment of actin Proc Natl Acad Sci USA 2004 101 10166 10171 10.1073/pnas.0402829101 15199184 PMC454183

[B3] Dehoux P Flores R Dauga C Zhong G Subtil A Multi-genome identification and characterization of chlamydiae-specific type III secretion substrates: the Inc proteins BMC Genomics 2011 12 109 10.1186/1471-2164-12-109 21324157 PMC3048545

[B4] Hackstadt T Fischer ER Scidmore MA Rockey DD Heinzen RA Origins and functions of the chlamydial inclusion Trends Microbiol 1997 5 288 293 10.1016/S0966-842X(97)01061-5 9234512

[B5] Grieshaber SS Grieshaber NA Hackstadt T Chlamydia trachomatis uses host cell dynein to traffic to the microtubule-organizing center in a p50 dynamitin-independent process J Cell Sci 2003 116 3793 3802 10.1242/jcs.00695 12902405

[B6] Geisler WM Suchland RJ Rockey DD Stamm WE Epidemiology and clinical manifestations of unique Chlamydia trachomatis isolates that occupy nonfusogenic inclusions J Infect Dis 2001 184 879 884 10.1086/323340 11528595

[B7] Ridderhof JC Barnes RC Fusion of inclusions following superinfection of HeLa cells by two serovars of Chlamydia trachomatis Infect Immun 1989 57 3189 3193 2550371 10.1128/iai.57.10.3189-3193.1989 PMC260788

[B8] Fields KA Fischer E Hackstadt T Inhibition of fusion of Chlamydia trachomatis inclusions at 32 degrees C correlates with restricted export of IncA Infect Immun 2002 70 3816 3823 10.1128/IAI.70.7.3816-3823.2002 12065525 PMC128059

[B9] Hackstadt T Scidmore-Carlson MA Shaw EI Fischer ER The Chlamydia trachomatis IncA protein is required for homotypic vesicle fusion Cell Microbiol 1999 1 119 130 10.1046/j.1462-5822.1999.00012.x 11207546

[B10] Howard L Orenstein NS King NW Purification on renografin density gradients of Chlamydia trachomatis grown in the yolk sac of eggs Appl Microbiol 1974 27 102 106 4855645 10.1128/am.27.1.102-106.1974 PMC379975

[B11] Scidmore MA Cultivation and Laboratory Maintenance of Chlamydia trachomatis Curr Protoc Microbiol 2005 Chapter 11 Unit 11A 1 10.1002/9780471729259.mc11a01s00 18770550

[B12] Askham JM Vaughan KT Goodson HV Morrison EE Evidence that an interaction between EB1 and p150(Glued) is required for the formation and maintenance of a radial microtubule array anchored at the centrosome Mol Biol Cell 2002 13 3627 3645 10.1091/mbc.E02-01-0061 12388762 PMC129971

[B13] Sharp GA Osborn M Weber K Ultrastructure of multiple microtubule initiation sites in mouse neuroblastoma cells J Cell Sci 1981 47 1 24 7021567 10.1242/jcs.47.1.1

[B14] Knowlton AE Brown HM Richards TS Andreolas LA Patel RK Grieshaber SS Chlamydia trachomatis infection causes mitotic spindle pole defects independently from its effects on centrosome amplification Traffic 2011 12 854 866 10.1111/j.1600-0854.2011.01204.x 21477082 PMC3116664

[B15] Suchland RJ Rockey DD Bannantine JP Stamm WE Isolates of Chlamydia trachomatis that occupy nonfusogenic inclusions lack IncA, a protein localized to the inclusion membrane Infect Immun 2000 68 360 367 10.1128/IAI.68.1.360-367.2000 10603409 PMC97142

[B16] Suchland RJ Jeffrey BM Xia M Bhatia A Chu HG Rockey DD Stamm WE Identification of concomitant infection with Chlamydia trachomatis IncA-negative mutant and wild-type strains by genomic, transcriptional, and biological characterizations Infect Immun 2008 76 5438 5446 10.1128/IAI.00984-08 18852248 PMC2583591

[B17] Schramm N Wyrick PB Cytoskeletal requirements in Chlamydia trachomatis infection of host cells Infect Immun 1995 63 324 332 7806372 10.1128/iai.63.1.324-332.1995 PMC172995

[B18] GORDON FB QUAN AL Occurence of glycogen in inclusions of the psittacosis-lymphogranuloma venereum-trachoma agents J Infect Dis 1965 115 186 196 10.1093/infdis/115.2.186 14308365

[B19] Fan VS Jenkin HM Glycogen metabolism in Chlamydia-infected HeLa-cells J Bacteriol 1970 104 608 609 5473914 10.1128/jb.104.1.608-609.1970 PMC248254

[B20] Russell M Darville T Chandra-Kuntal K Smith B Andrews CW O’Connell CM Infectivity acts as in vivo selection for maintenance of the chlamydial cryptic plasmid Infect Immun 2011 79 98 107 10.1128/IAI.01105-10 20974819 PMC3019909

[B21] Rockey DD Fischer ER Hackstadt T Temporal analysis of the developing Chlamydia psittaci inclusion by use of fluorescence and electron microscopy Infect Immun 1996 64 4269 4278 8926099 10.1128/iai.64.10.4269-4278.1996 PMC174367

[B22] Scidmore-Carlson MA Shaw EI Dooley CA Fischer ER Hackstadt T Identification and characterization of a Chlamydia trachomatis early operon encoding four novel inclusion membrane proteins Mol Microbiol 1999 33 753 765 10.1046/j.1365-2958.1999.01523.x 10447885

