# Supplementary figures and images for: Chlamydia trachomatis homotypic inclusion fusion is promoted by host microtubule trafficking
Source: BMC Microbiol. 2013 Aug 7;13:185. doi: 10.1186/1471-2180-13-185 (PMC3750546; doi:10.1186/1471-2180-13-185)

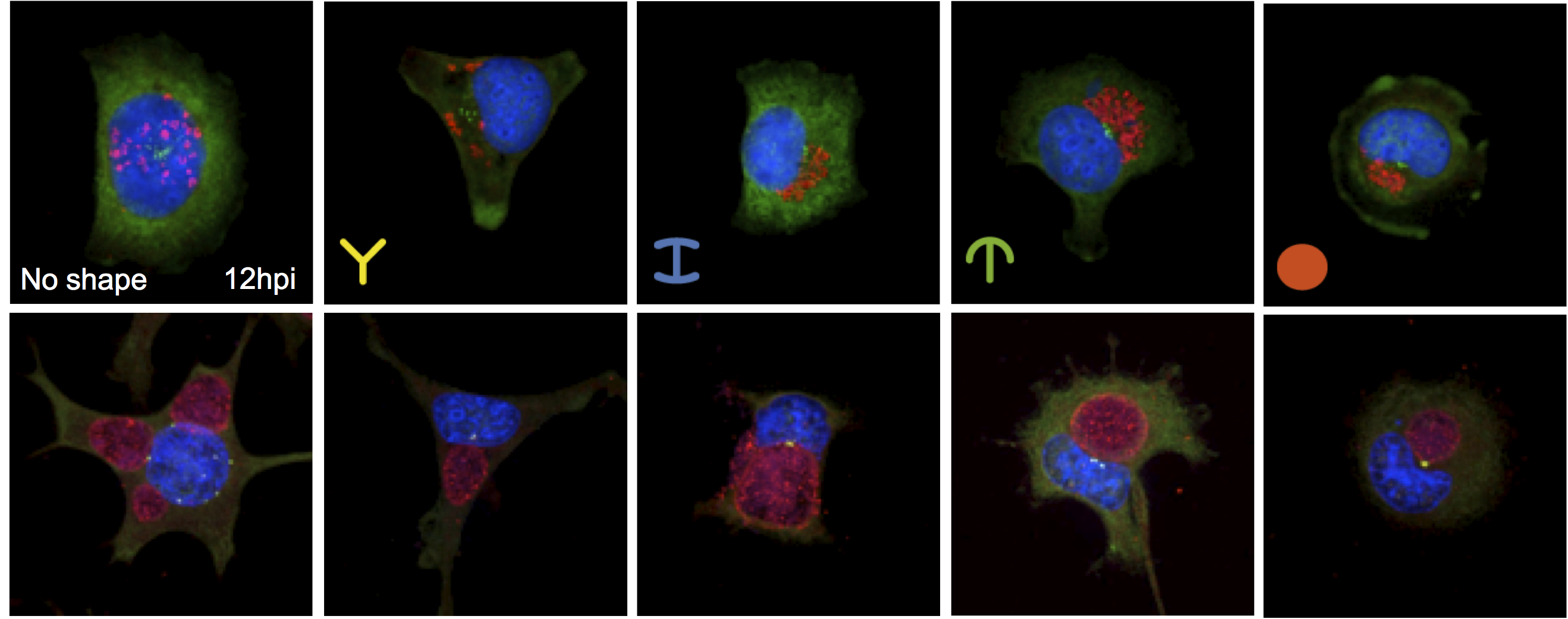

Supplement: Additional file 2: Figure 2 — Centrosome positioning affects chlamydial inclusion localization. Uninfected and infected neuroblastomas were plated on CYTOOchips (glass coverslips imprinted with fibronectin micropatterns). Each micropattern is indicated in the lower left of the top panel. Infected cells were fixed at 12 and 24 hpi (top and bottom panel for each shape, respectively). Cells were stained with antibodies to g-tubulin (green) and Chlamydia (red). Nucleic acid is visualized by staining with DRAQ5 (blue). [file 1471-2180-13-185-S2.tiff]
